# Surface Performance Evaluation and Mix Design of Porous Concrete with Noise Reduction and Drainage Performance

**DOI:** 10.3390/ma18235433

**Published:** 2025-12-02

**Authors:** Yijun Xiu, Miao Hu, Chenlong Zhang, Shaoqi Wu, Mulian Zheng, Jinghan Xu, Xinghan Song

**Affiliations:** 1 Key Laboratory for Special Area Highway Engineering of Ministry of Education, Chang’an University, Xi’an 710064, China; 18588852713@163.com (Y.X.); humiao@chd.edu.cn (M.H.); wushaoqi1021@163.com (S.W.); 2021021051@chd.edu.cn (J.X.); songxinghan@chd.edu.cn (X.S.); 2 Guangdong Road and Bridge Construction Development Co., Ltd., Guangzhou 510635, China; zhchl1987@126.com

**Keywords:** porous concrete pavement, PCNRD, sound absorption performance, drainage performance, anti-wear performance, mix design method

## Abstract

Porous concrete is widely recognized as an eco-friendly pavement material; however, existing studies mainly focus on its use as a base course, and systematic investigations on porous concrete specifically designed for heavy-traffic pavements and multifunctional surface performance remain limited. In this study, a novel multifunctional porous concrete with integrated noise reduction and drainage performance (PCNRD) was developed as a top-layer pavement material, addressing the performance gap in current applications. A comprehensive evaluation of the surface properties of porous concrete was performed based on tests of the sound absorption, void ratio, permeability, and wear resistance. The results demonstrate that the porous concrete exhibits excellent sound absorption (sound absorption coefficient 0.22–0.35) and high permeability (permeability coefficient 0.63–1.13 cm/s), and superior abrasion resistance (abrasion loss ≤ 20%) within an optimized porosity range of 17–23%. Furthermore, an optimized pavement thickness (8–10 cm) was proposed, and functional correlations among key surface performance indicators were revealed for the first time. Based on a uniform experimental design, four key mix parameters (water–cement ratio, cement content, silica fume content, and cement strength grade) were examined using strength and effective porosity as dual control indices, leading to the development of a novel mix design method tailored for PCNRD. This study not only fills the technical gap in high-performance porous concrete for heavy-traffic pavement surfaces but also provides a practical scientific framework for its broader engineering application.

## 1. Introduction

To establish a livable and sustainable residential environment, it is essential to mitigate various environmental issues such as air pollution, water and soil contamination, and noise pollution. Among these, traffic noise has emerged as a particularly pressing concern. To address this challenge, the advancement of environmental standards and road engineering technologies has led to growing interest in the application of porous concrete for pavement surfaces [1]. Porous concrete demonstrates excellent permeability to both air and water, as well as superior sound absorption, owing to its continuous porosity achieved through the use of uniformly graded coarse aggregates [2,3]. Porous, or pervious, concrete is composed primarily of coarse aggregates bound by a cementitious matrix, within which continuous voids are deliberately introduced. Due to its high permeability, favorable acoustic performance, and other beneficial attributes, it is widely recognized as an environmentally sustainable material [4,5].

In recent years, researchers worldwide have systematically investigated the mechanical behavior, durability, drainage capacity, noise reduction performance, and mix design optimization of porous concrete [6,7,8,9,10,11]. The fundamental characteristics of this material have been extensively documented in the literature [3,12,13,14,15]. Nevertheless, conventional materials and traditional mix design methods often yield porous concrete with insufficient strength. Consequently, its use has been largely restricted to light-duty applications such as parking areas, pedestrian walkways, and bicycle paths. Studies have shown that incorporating silica fume (SF) and superplasticizers (SPs) can substantially enhance the strength of porous concrete [6], enabling it to withstand heavier traffic loads through the addition of suitable admixtures. M.L. Zheng et al. [14] investigated the permeable base of porous concrete, proposing that the effective particle size and uniformity coefficient serve as reliable indicators of aggregate gradation in mix design. M. Ziccarelli [5] discusses the mix design of pervious concrete in geotechnical engineering applications, indicating that an appropriate porosity can significantly enhance the concrete’s engineering performance, particularly in sound absorption and drainage. Y. Zhang and X.J. Xu et al. [16] developed a Gaussian Process Regression (GPR) model to predict multiple properties of permeable concrete based on key mix parameters, such as the nominal coarse aggregate size, cement content, water–cement ratio, and coarse aggregate content. This model enables the rapid and low-cost evaluation of permeable concrete performance. B. Debnath and P.P. Sarkar et al. [17] investigated the use of over-burnt brick as coarse aggregate in pervious concrete, focusing on its mechanical properties and pore characteristics, and developed predictive equations for porosity, permeability, and compressive strength.

Research indicates that the porosity, pore morphology, and material thickness of porous concrete significantly influence its noise reduction performance [18,19,20]. P. Mikhailenko, Z.Y. Piao et al. [2] reviewed low-noise pavement technologies, highlighting that a porosity range of 15–30% is optimal for effective noise absorption. Zheng et al. suggested that, to meet drainage demands, the permeability coefficient of porous concrete should exceed 1.05 cm/s, with an effective porosity maintained between 20% and 30%. Optimal noise reduction is typically achieved when target porosity ranges between 15% and 30% [21,22]. C. Sánchez-Mendieta et al. noted in their review that, to achieve a balance between high permeability and sufficient strength, the recommended mix design for porous cementitious concrete should use aggregate sizes ranging from 4.5 to 20 mm, considering both fine aggregate and fine-aggregate-free systems [23]. F.T. Lu et al. [24] optimized the mix design of permeable concrete, finding that while increasing the target porosity enhances permeability, it reduces the compressive strength and tensile strength. G.O. Claudino, G.G.O. Rodrigues et al. [8] optimized the mix design for pervious concrete by balancing the cement paste and granular skeleton, enhancing both the mechanical strength and permeability while improving the sound absorption performance. Z.Q. Wang, J.G. Xie et al. [21] presented a sound absorption model for porous asphalt concrete based on the permeability coefficient, showing that porosity and permeability are closely related and significantly affect the sound absorption performance. G.L. Qu, M.L. Zheng et al. [13] employed a multi-objective optimization approach based on the RSM-MOPSO-GA algorithm to enhance the performance of high-performance porous concrete, highlighting the synergistic enhancement of porosity and other design parameters to improve overall concrete performance. Drainage performance, governed by pore connectivity, must simultaneously satisfy mechanical strength requirements. The cement content, water-to-binder ratio, and aggregate gradation are all critical factors influencing permeability [25,26,27,28].

Although significant progress has been made in understanding the fundamental properties of porous concrete, notable limitations remain [29,30,31]. Most existing studies examine individual functional properties—such as permeability or acoustic absorption—without establishing a comprehensive framework that simultaneously integrates noise reduction, drainage capacity, and abrasion resistance [32,33,34,35]. Moreover, experimental investigations specifically targeting porous concrete designed as the top pavement layer for heavy-traffic applications are still scarce, and the development of systematic mix design methodologies for multifunctional porous concrete remains limited [36,37]. To address these gaps, this study develops a novel noise-reducing and drainable porous concrete (PCNRD) tailored for pavement surface applications and conducts a holistic evaluation of its functional surface performance, including noise reduction, drainage capability, and wear resistance. A comprehensive experimental program—consisting of the standing wave tube method, volumetric porosity measurement, permeability testing, and the Cantabro abrasion test—was carried out to characterize the surface behavior of PCNRD. Furthermore, based on uniform experimental design principles, functional correlations among key performance indicators were established for the first time, and a new mix design procedure specific to PCNRD was proposed using strength and effective porosity as dual control parameters. The outcomes of this research not only fill an important technical gap in the study of multifunctional porous concrete for heavy-traffic pavement surfaces but also provide a scientifically grounded and practically applicable framework for promoting its broader implementation, thereby contributing to the advancement of sustainable and environmentally friendly pavement systems.

## 2. Materials and Methods

### 2.1. Experimental Design

#### 2.1.1. Materials

The porous concrete specimens were prepared using cement, coarse aggregates, silica fume, a water-reducing admixture and water. The coarse aggregate used was diorite. The properties of the raw materials and their corresponding proportions are presented in Table 1. The particle size distribution of the coarse aggregates is shown in Table 2. This study utilized silica fume provided by Gansu Sanyuan Silica Fume Co., Ltd. (Lanzhou, China) The chemical composition of silica fume is provided in Table 3. This study employs P.O.42.5 ordinary Portland cement from the Qin ling Mountains in Shaanxi Province. The main chemical composition of the cement is listed in Table 4. The water-reducing admixture was supplied by Henan Xinglong Chemical Co., Ltd. (Zhengzhou, China). The water used in this study had no special requirements other than being clean and free of impurities. Tap water was used throughout the experiments.

#### 2.1.2. Specimen Preparation

The fabrication of porous cementitious concrete specimens comprises three sequential stages. The first stage involves mixing, wherein the distinctive characteristics of porous concrete render the order of material incorporation and the duration of blending particularly critical. Initially, cement, silica fume, and coarse aggregates are dry-blended for 30 s. Subsequently, water and a water-reducing admixture are introduced, followed by a 90 s wet mixing process. The resulting mixture is then cast into the designated molds.

The second stage concerns molding. In contrast to conventional cement concrete, the uniformity of paste encapsulation around the aggregates and the degree of inter-aggregate compaction exert a pronounced influence on both mechanical performance and pore structure. Consequently, traditional concrete forming procedures are unsuitable for this material system. In the present study, an overhead vibration compaction technique was adopted, employing a vibrator operating at 50 Hz with an excitation force of 5 kN for a duration of 30 s.

The third stage is curing. Owing to the extensive network of interconnected voids, freshly formed porous cement concrete is susceptible to accelerated moisture loss, which compromises hydration. To mitigate this effect, specimens were immediately enveloped in plastic film upon molding. After 24 h, the specimens were demolded and transferred to a controlled curing environment. Standard curing was performed at a temperature of 20 ± 3 °C and a relative humidity not less than 90%.

All specimens were prepared under the conditions delineated in Table 5 and Table 6. The void ratio was quantified using standardized test specimens. The specimens were molded under the conditions specified in Table 5 and Table 6. The void ratio was determined using standard test specimens.

### 2.2. Testing Method

#### 2.2.1. Sound Absorption Test

**Figure 1:**
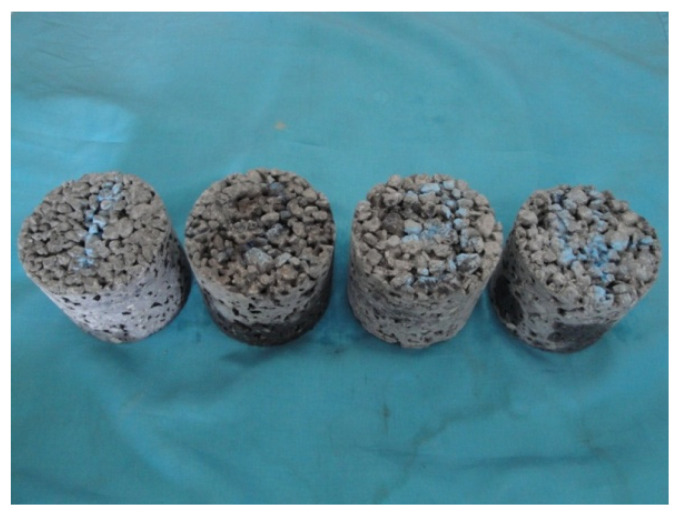
Specimens of different cement contents.

**Figure 2:**
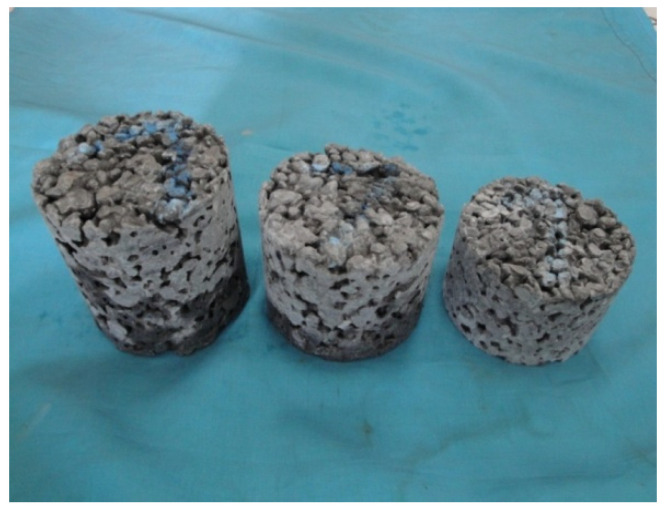
Specimens of different thicknesses.

The sound absorption test was conducted in accordance with the Chinese national standard GBJ88-85, Measurement Specifications for the Standing Wave Tube Sound Absorption Coefficient and Acoustic Impedance Ratio. To investigate the influence of mix proportions on the acoustic absorption properties of porous concrete, cylindrical specimens with a diameter of 95 mm and a height of 80 mm were prepared, as illustrated in Figure 1. The corresponding mix proportions are presented in Table 4. Specimens with varying thicknesses are shown in Figure 2, with an effective porosity of approximately 19%.

**Figure 3:**
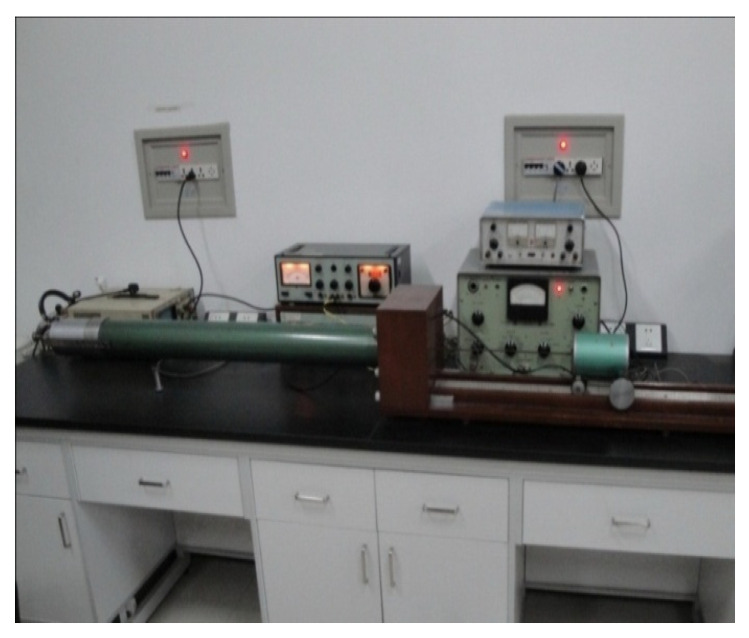
Sound absorption device.

**Figure 4:**
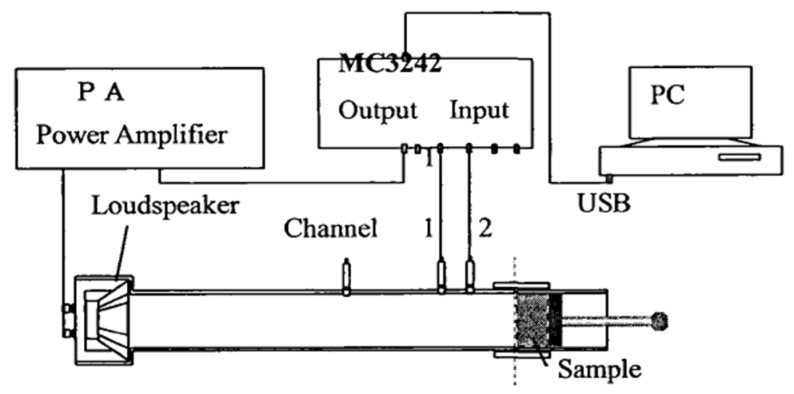
Experimental principle of sound absorption test.

The standing wave tube apparatus (Figure 3 and Figure 4) is employed to measure the sound absorption coefficient under conditions of normal sound wave incidence. The testing procedure is as follows:

(1) Prepare cylindrical specimens with a diameter of 95 mm, as specified in Table 4.
(2) Place the specimens into the test apparatus and seal all gaps with plasticine to ensure airtightness.
(3) Position the apparatus within the testing device, adjust the frequency, and record the corresponding sound absorption coefficients.

The sound absorption coefficient is calculated as shown in Equations (1) and (2).

(1) $$\alpha =\frac{E_{\alpha }}{E_{i}}$$

(2) $$E_{\alpha }=E_{i}-E_{r}$$

where α is the sound absorption coefficient of the material, $E_{\alpha }$ is the acoustic energy absorbed by material or structure, $E_{i}$ is the total energy on the incident material or structure, and $E_{r}$ is the acoustic energy reflected by the material or structure. 

Given that the thickness of porous concrete pavement is considerably smaller than the acoustic wavelength, the effects of air thermal conductivity and viscosity can be disregarded. As the acoustic impedance of air is minimal and the porous material itself cannot resonate with sound waves, it is treated as an acoustically rigid medium. In this study, PCNRD is applied to road sections, such as tunnel entrances, curves, bridge decks, parking areas, ascending and descending ramps, intersections, and highway on- and off-ramp zones, typically characterized by low vehicle speeds. Consequently, the dominant frequency range of tire–road noise in these areas lies between 400 and 1000 Hz. Accordingly, the incident acoustic wave frequency was controlled within the range of 125 to 2000 Hz, with sound absorption coefficients measured at intervals of 50 Hz.

#### 2.2.2. Void Ratio Test

From a drainage perspective, voids are classified into effective and ineffective voids. Effective voids are those that allow water to pass through and be expelled. From a water flow dynamics perspective, only interconnected voids that are not occupied by bound water are considered effective. Water in semi-connected voids is relatively stagnant and ineffective from the standpoint of water movement, but it can gradually drain out, making these voids effective for drainage purposes. Therefore, in drainage performance assessments, effective porosity includes both connected and semi-connected pores, while closed voids are classified as ineffective. The total void ratio is the percentage of total void volume relative to the aggregate mixture, commonly referred to as the void ratio. The effective void ratio is the percentage of effective void volume relative to the total volume of the mixture. In the context of analyzing the drainage performance of porous cement concrete, the effective void ratio is a simple, efficient, and effective evaluation metric.

The void ratio was determined using the volumetric method, and the testing procedure was conducted as follows:

(1) The specimen’s length, width, and height were measured three times, and the average values were calculated.
(2) The specimen volume was then computed, and the porosity was determined using Equations (3) and (4).

(3) $$n_{0}=\left(1-\frac{\rho_{s}}{\rho_{t}}\right)\times 100$$

(4) $$n_{e}=\left(1-\frac{m_{2}-m_{1}}{v\cdot \rho_{w}}\right)\times 100$$

where $n_{0}$ is the full porosity of the specimen (%), $n_{e}\;$ is the effective porosity of the specimen (%), $\rho_{s\;}$ is the bulk density of the porous cement concrete (g/cm^3^), $\rho_{t}$ is the theoretical density of the porous cement concrete (g/cm^3^), $m_{1}$ is the specimen mass after soaking for 24 h (g), $m_{2}$ is the specimen mass after drying for 24 h in a 60 °C oven (g), ν is the specimen volume measured by amount volume method (cm^3^), and $\rho_{w}$ is the density of water (g/cm^3^).

During the cement hydration process, approximately one-quarter of the mixing water chemically combines with cement to form bound water in a stable state [38,39,40]. At this stage, the bound water undergoes volumetric contraction, with its mass accounting for roughly 25% of the original water content. In addition to the chemically bound water, the remaining free water facilitates complete cement hydration and eventually evaporates into the atmosphere. Based on the foregoing analysis, Equation (5) can be derived to calculate the theoretical density of porous concrete:

(5) $$\rho_{t}=\frac{100+P_{c}+P_{c}\times \frac{1}{4}}{\frac{100}{\rho }+\frac{P_{c}}{\rho_{c}}+P_{c}\times \frac{1}{4}\times \frac{3}{4}}\times \rho_{w\;}$$

where $\rho_{t}\;$ is the theoretical density of porous concrete (g/cm^3^); $P_{c}\;$ is the mass ratio of cement and aggregate (%); $\rho_{c}$ is the relative density of cement, which is 3.1 g/cm^3^; ρ is the apparent density of aggregate (g/cm^3^); $\rho_{w}$ is the density of water, which is 1 g/cm^3^.

#### 2.2.3. Permeability Coefficient Test

Darcy’s law is applicable only under laminar flow conditions, with the flow regime being directly influenced by the hydraulic gradient. According to AASHTO T-215 and ASTM 2434-74, materials with low compactness exhibit hydraulic gradient values ranging between 0.2 and 0.3, whereas highly compacted materials maintain laminar flow when the gradient lies between 0.3 and 0.5. In Japanese standards (JIS A1218T-1979), the upper limit of the hydraulic gradient for sand is specified as 0.3. The flow regime is closely associated with both the void ratio and pore size. To investigate the relationship between the effective porosity and the permeability coefficient, specimens with varying porosities were prepared. The permeability coefficient tests were conducted at a controlled water temperature of 10 °C.

**Figure 5:**
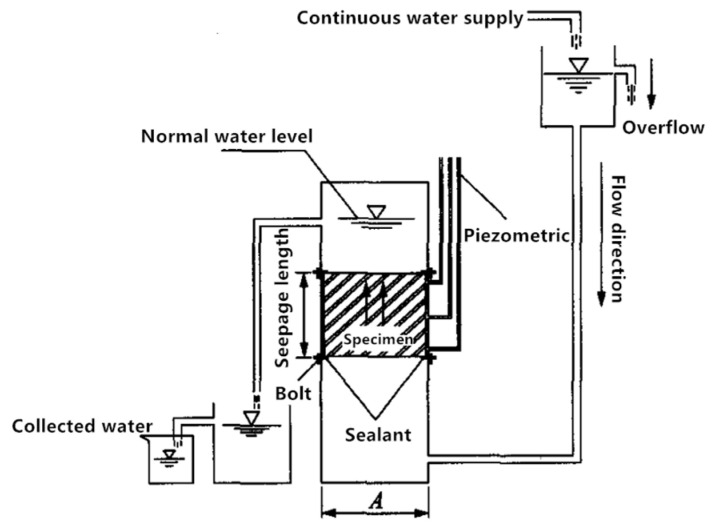
Permeability coefficient tester schematic.

This research group developed a permeability coefficient tester, as shown in Figure 5, to determine the hydraulic conductivity of porous materials. The permeability coefficient, also referred to as hydraulic conductivity, serves as a key indicator of a material’s capacity to transmit fluids. Current studies on the permeability of large-pore materials are primarily based on the principles of Darcy’s law, as expressed in Equations (6)–(8).

(6) $$q=kA\frac{\left(h_{1}-h_{2}\right)}{L}$$

(7) v=k×i

(8) $$i=\frac{\left(h_{1}-h_{2}\right)}{L}$$

In the constant-head permeability test, the hydraulic head difference between the two ends of the specimen remains constant, and thus the hydraulic gradient is fixed. Accordingly, Equation (6) can be reformulated as Equation (9).

(9) q/t=k×i×A

The permeability coefficient can be determined using Equation (10).

(10) $$k=\frac{qL}{At\Delta h}$$

where q is the volume of flow within *t* seconds (cm^3^), A is the cross-sectional area (shown in Figure 5) (cm^2^), Δh  is the head difference (=$h_{1}-h_{2}$) (cm), L  is the seepage length (as shown in Figure 5) (cm), ν is the seepage rate (cm/s), k is the permeability coefficient, ⅈ  is the hydraulic gradient, and *t* is the seepage time (s).

#### 2.2.4. Wear Resistance Experiment

Unlike conventional concrete, the aggregate gradation of porous concrete primarily consists of coarse aggregates ranging from 5 to 10 mm and 10 to 16 mm in size. Due to the inherently rough surface texture of porous concrete pavements, traditional testing methods are unsuitable for this application.

The Cantabro test of asphalt mixtures can be used to evaluate the optimum asphalt content, which characterizes the degree of aggregate off the road surface and dissipate due to the amount of asphalt or insufficient adhesion under traffic load. Porous concrete drainage material is similar to the large-size asphalt mixture in the composition of the aggregate size [41,42]. So, using the Cantabro test to evaluate its anti-wear performance can effectively characterize the cohesiveness between the aggregate and cement paste. It also can reflect the resistance capacity of porous concrete pavement aggregate loss under the effect of abrasion and impact.

The cement content directly affects the adhesive bond between the aggregate and the cementitious matrix, thereby enhancing the material’s resistance to abrasion and impact induced by vehicular loading [43]. The abrasion loss of porous concrete can be determined using Equation (11). The fundamental testing procedures were conducted in accordance with the Standard Test Methods of Bitumen and Bituminous Mixtures for Highway Engineering of China (JTG E20-2011).

(11) $$∆s=\frac{m_{0}-m_{1}}{m_{0}}\times 100$$

where Δs  is the abrasion loss of porous concrete (%), $m_{0}$ is the initial mass of the specimen (g), and $m_{1}\;$ is the mass of the specimen after abrasion (g).

The basic information of the specimen for the anti-wear test is shown in Table 4. Moreover, this article adopts the method of upper vibration molding, and the size of the specimen is the same as the standard specimen used in the Marshall test. The arithmetic mean of attrition loss of the specimen is the final test result. When the loss of a specimen is more than 15% of the average value, the data of the specimen should be removed, and the average of the remaining two test data sets is the outcome of the test result. If the abrasion loss of two specimens is more than 15% of the average value, the test data should be re-tested.

#### 2.2.5. Strength Test

The mechanical property tests were performed in accordance with ASTM C78 (ASTM Standard C78, 2002) and the Test Methods of Cement and Concrete for Highway Engineering (JTG E30-2005) of China.

#### 2.2.6. Workability Evaluation Test

Based on the test method for determining the mass per unit volume and air content of fresh concrete outlined in the Construction Guide of Cement Concrete Pavement of Japan (JIS A116), the following workability evaluation procedure is proposed.

(1) Test Procedure

Place the fresh concrete into a designated container in three successive layers, compacting each layer by vibrating it with a poker 25 times. After filling, invert the container onto a flat surface and carefully remove it. Observe the condition of the remaining specimen on the flat surface.

(2) Evaluation of Workability

The workability of fresh concrete is classified as shown in Table 7. The appropriate consistency of the cement paste is essential to achieve satisfactory workability in PCNRD. The amount of cement paste should be slightly excessive after the surface of each coarse aggregate particle is fully coated and a stable cementitious film has formed. A modest surplus of cement paste enhances the workability of PCNRD, provided that the slurry does not flow out and the coarse aggregates remain fully encapsulated without disintegration.

Grades A and B indicate an overly stiff mixture, where the cement paste fails to completely coat the aggregate surfaces, resulting in poor cohesion. Grades D and E denote an excessively fluid mix, where the slurry is overly mobile and adhesive strength is inadequate. Grade C represents an optimal and satisfactory workability condition. This evaluation method was employed in the present study to verify the workability of the mix design.

### 2.3. Mix Design Parameters

Factors such as the water–cement ratio, cement content, gradation, cement strength grade, and admixture dosage exert a significant influence on the pavement performance of PCNRD. The aggregate gradation is further elaborated upon in subsequent analyses. In this uniform experimental design, four principal factors—namely, the water–cement ratio, cement content, cement strength grade, and admixture content—were selected. The factor levels are presented in Table 8. The uniform design method was adopted, and the mixture ratios were determined as shown in Table 9.

## 3. Results and Discussion

### 3.1. Sound Absorption Performance

#### 3.1.1. The Influence of Mix Proportion on Sound Absorption Performance

**Figure 6:**
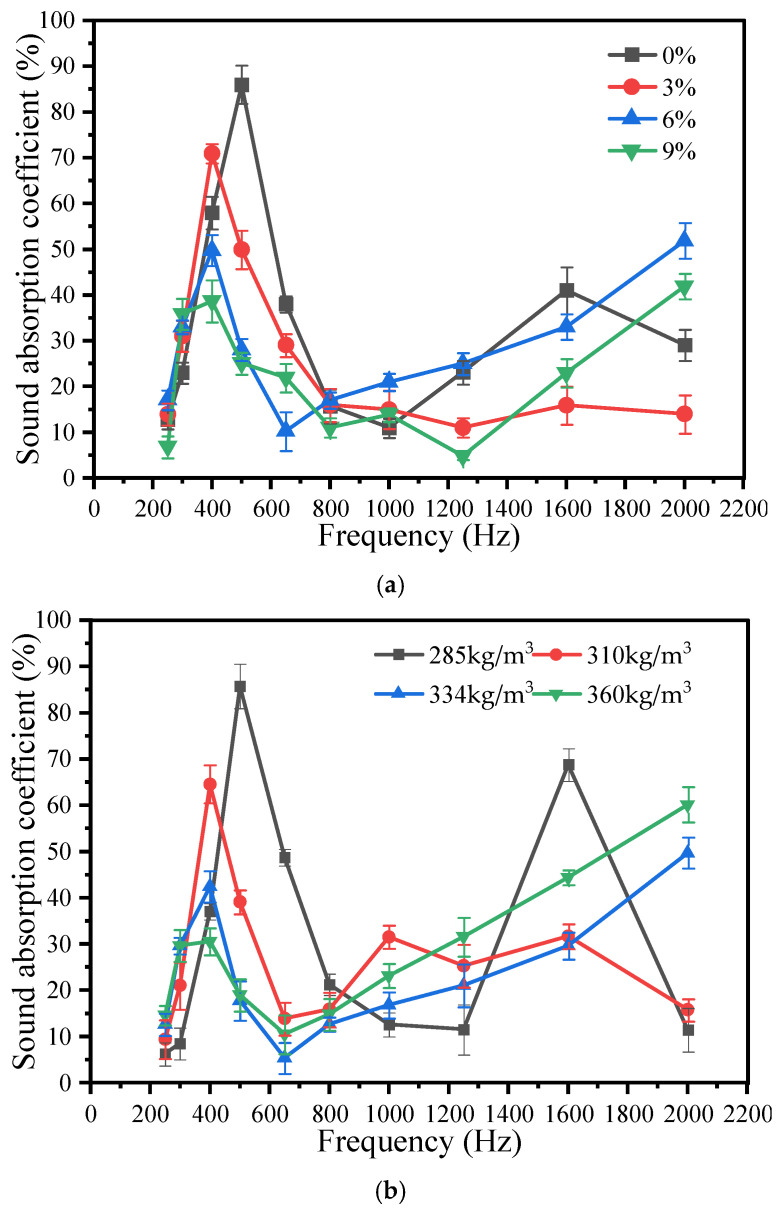
Effect of mix proportion on the sound absorption coefficient of porous concrete: (**a**) different silica fume contents (%); (**b**) different cement contents (kg/m^3^).

As illustrated in Figure 6, the frequency range in which the sound absorption coefficient exceeds 20% is primarily concentrated between 400–800 Hz and 1600–2000 Hz, while a distinct trough appears near 1000 Hz. The noise generated by low-speed vehicles generally falls within 400–1000 Hz; thus, incorporating lower dosages of silica fume and cement can effectively enhance the sound absorption capacity of the material.

The void ratio varies with changes in the mix proportion, as demonstrated in Table 10. In practical engineering applications, the average of the sound absorption coefficients at the four octave band center frequencies—250, 500, 1000, and 2000 Hz—is typically used to characterize the acoustic performance of a material. This average, referred to as the sum average, serves as a criterion: when its value exceeds 0.2, the material is classified as sound-absorbing; otherwise, it is considered sound-reflecting. As shown in Table 6, all sum average values surpass 0.2, thereby confirming that porous concrete possesses distinct sound-absorbing properties. Table 10 also presents the arithmetic mean of the sound absorption coefficients across all tested frequencies.

The correlation between the void ratio and sound absorption coefficient is further illustrated in Figure 6 and Table 10. An increase in the void ratio results in a higher peak of the sound absorption coefficient. With more internal pores, incident acoustic waves undergo prolonged diffuse reflection within the concrete structure, during which acoustic energy is dissipated through continuous friction between the wave and the inner pore walls. Consequently, a greater portion of the acoustic energy is absorbed, effectively reducing noise levels.

Figure 6 also reveals that the range of frequencies corresponding to a sound absorption coefficient of ≥20% expands with an increasing void ratio. In other words, larger pores enable the material to absorb sound across a broader frequency spectrum, thereby enhancing its overall noise reduction performance.

**Figure 7:**
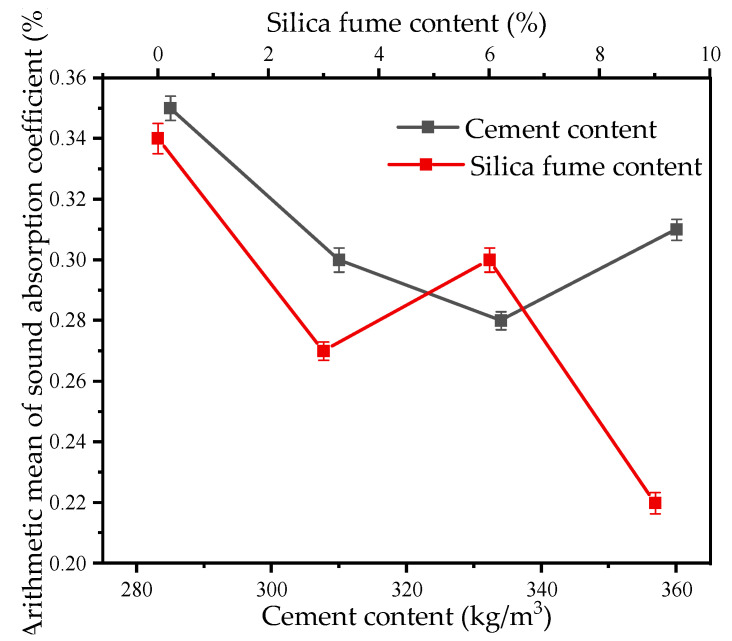
Arithmetic mean of sound absorption coefficient under different mix proportions.

**Figure 8:**
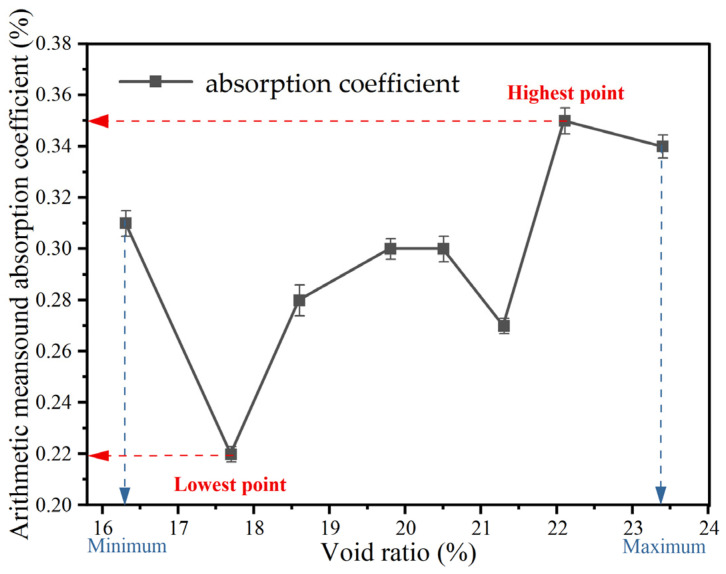
Arithmetic mean of sound absorption coefficient under different void ratios.

The arithmetic mean curves of the sound absorption coefficient for different mix proportions and void ratios are illustrated in Figure 7 and Figure 8. These curves clearly indicate that a higher void ratio corresponds to a higher arithmetic mean sound absorption coefficient. The arithmetic mean values range from 0.22 to 0.35, while the associated void ratios fall between approximately 17% and 23%, satisfying the acoustic requirements for effective sound absorption.

#### 3.1.2. The Effect of Thickness on Sound Absorption Performance

**Figure 9:**
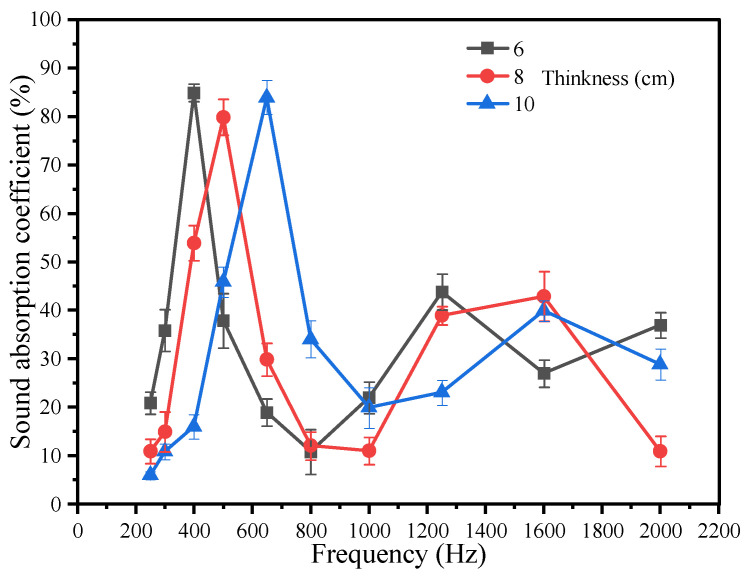
Impact of thickness on the sound absorption properties.

The sound absorption coefficient of specimens with varying thicknesses exhibits a discernible shift toward higher frequency ranges, as illustrated in Figure 9. The peak sound absorption coefficient transitions from 400 Hz to 630 Hz with an increasing specimen thickness, while its magnitude remains relatively stable within the 1250–2000 Hz frequency band. Consequently, as the thickness of the porous concrete increases, the corresponding sound absorption range progressively shifts toward higher frequencies.

When the specimen thicknesses are 6 cm, 8 cm, and 10 cm, the principal sound absorption ranges are 315–500 Hz, 400–630 Hz, and 500–800 Hz, respectively. The frequency spectrum of road traffic noise, typically between 400 and 1000 Hz, aligns more closely with the absorption ranges observed for thicknesses of 8 cm and 10 cm. From this perspective, these two thicknesses exhibit superior acoustic performance. However, the final determination of thickness should comprehensively consider mechanical properties and structural requirements.

In permeable/porous concrete or high-void pavement materials, the peak frequency of the sound absorption coefficient shifts toward higher frequencies as the thickness increases [43]. Based on the findings of our group, the results indicate that an upper-layer thickness within the range of 8–10 cm provides satisfactory mechanical performance while maintaining stable functional behavior.

### 3.2. Drainage Performance

#### 3.2.1. Relationship Between Full Porosity and Effective Porosity

**Figure 10:**
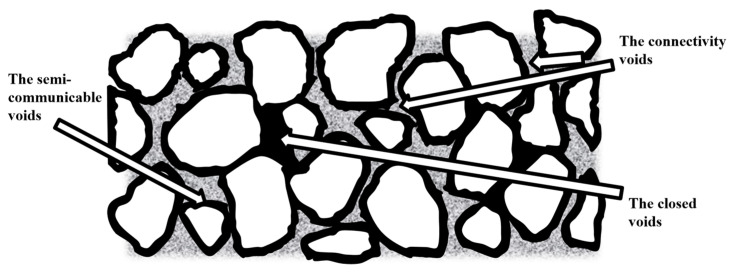
Schematic diagram of the internal voids of porous concrete.

Effective porosity serves as a simple, practical, and reliable indicator for assessing the drainage performance of porous concrete. The drainage capacity of the pavement is primarily governed by the effective porosity, which encompasses both connected and semi-connected voids, as illustrated in Figure 10. Therefore, it is essential to investigate the relationship between effective porosity and total porosity, with the latter encompassing all voids within the material.

**Figure 11:**
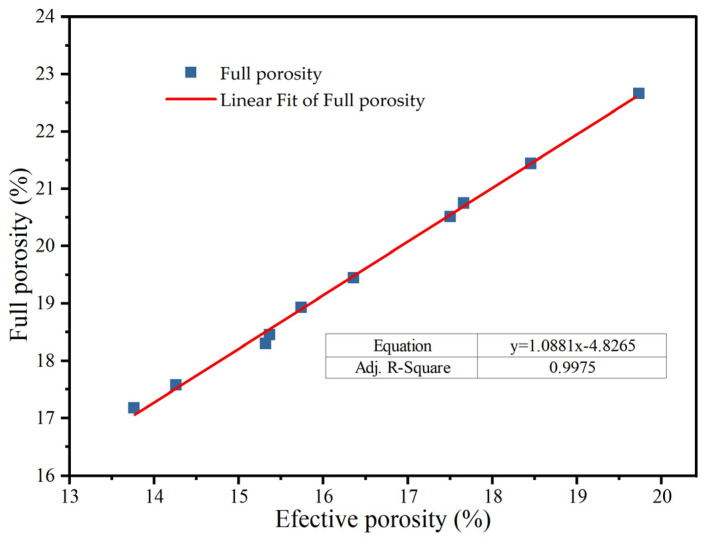
Relationship between effective porosity and full porosity.

According to Figure 11, the effective porosity and full porosity have a good linear correlation, which can be described by Equation (12). This linear relationship indicates that the effective porosity increases in direct proportion to the full porosity, suggesting that both parameters are closely linked in characterizing the material’s porosity. This correlation underscores the relevance of full porosity as a reliable predictor for effective porosity, which is crucial for evaluating the material’s drainage and sound absorption capabilities.

(12) $$n_{e}=1.0881n_{0}-4.8265(R^{2}=0.9975)$$

where $n_{e}$ is the effective porosity and $n_{0}$ is the full porosity.

#### 3.2.2. Relationship Between Full Porosity and Coarse Aggregate Content

**Figure 12:**
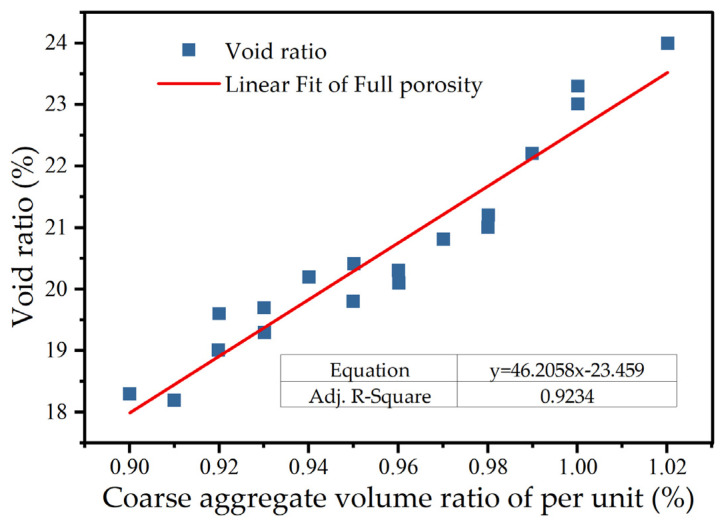
Relationship between coarse aggregate amount and void ratio.

In the mix proportion design of porous concrete, it is essential to ensure that the porosity remains relatively stable before and after mixing. Theoretically, when porous concrete specimens are produced under identical conditions—such as compaction and material composition—the variation in porosity is minimal. Research findings indicate that the quantity of coarse aggregate is the most critical factor influencing the porosity of concrete. Therefore, it is necessary to investigate the relationship between the total porosity and the amount of coarse aggregate, as shown in Figure 12. To simplify the mix design process, the coarse aggregate volume ratio per unit (α) is introduced to characterize the aggregate content, as expressed in Equation (13).

(13) $$\alpha =m_{g}∕\rho_{0g}$$

where $m_{g}\;$ is the coarse aggregate mass in 1 m^3^ of concrete and $\rho_{0g}$ is the compaction density.

The relationship between α and porosity, as expressed in Equation (14), was derived using the least-squares regression method. The correlation coefficient (*R*^2^) demonstrates that the relationship between the void ratio and coarse aggregate content exhibits a strong linear correlation. In materials science and engineering, the acceptable range of *R*^2^ values typically depends on the complexity of the material system and the interplay of multiple factors. For material systems like porous concrete, which exhibit nonlinear behavior influenced by several factors, the prevailing view in the literature is that [44] *R*^2^ ≥ 0.80 indicates a good fit, while *R*^2^ ≥ 0.90 is generally considered excellent and reflects a high fitting accuracy. The established α–porosity relationship (Equation (14)) and the strong linear correlation between the void ratio and coarse aggregate content (*R*^2^ = 0.9234) are statistically reliable.

(14) $$n_{0}=46.058\alpha -23.459(n=18,R^{2}=0.9234)$$

#### 3.2.3. Permeability Coefficient

**Figure 13:**
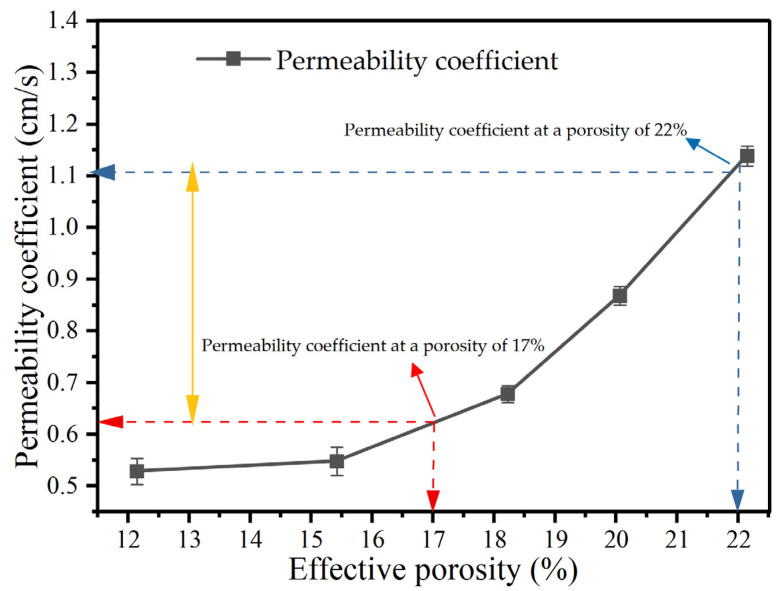
Effect of effective porosity on the permeability coefficient.

Figure 13 indicates a positive correlation between the porosity and permeability coefficient, consistent with recent systematic studies on the coupling of the pore structure and hydraulics in permeable concrete [45]. When the porosity ranges from 17% to 22%, the corresponding permeability coefficient of porous concrete varies from 0.63 to 1.13 cm/s. This is consistent with the latest measured regression results showing that 15–25% porosity corresponds to approximately 0.32–1.02 cm/s [46]. As shown in Figure 13, under conditions of equal porosity, the permeability coefficient of permeable concrete is typically significantly higher than that of porous asphalt. This finding aligns with multiple comparative conclusions regarding the pore size distribution and connectivity of the two materials, thereby validating the superior performance of permeable concrete as a drainage material and drainage pavement [47].

### 3.3. Anti-Wear Performance

**Figure 14:**
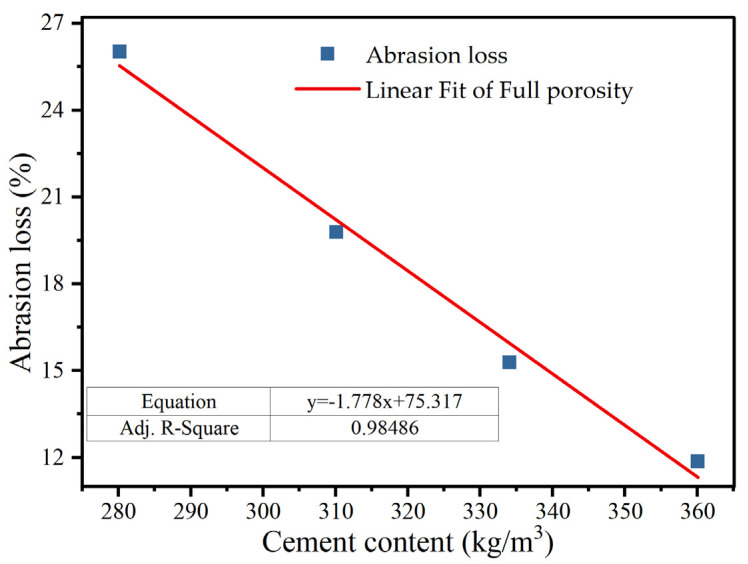
The relationship of abrasion loss and cement content of porous concrete.

As illustrated in Figure 14, the abrasion loss of porous concrete declines markedly with increasing cement content. This trend arises from the thickening of the cement paste layer enveloping the coarse aggregates as the cement dosage increases [7]. As a result, the interfacial bonding stress between the cement paste and coarse aggregates is enhanced, yielding a substantial improvement in wear resistance [26]. The empirical benchmark for mass loss in the asphalt Cantabro test is generally below 20% [47]. Based on current experimental observations and practical evidence, the abrasion loss of porous concrete materials likewise remains under 20%.

### 3.4. Mix Design

#### 3.4.1. Mix Design Indices

When designing the mix proportions for PCNRD, both mechanical strength and functional performance—specifically noise reduction and drainage capacity—must be ensured to meet the required standards.

(1) Strength

The mechanical strength of concrete directly determines the load-bearing capacity of the pavement. In the case of PCNRD, the presence of a highly porous structure results in a lower strength compared with conventional concrete, thereby limiting its applicability across different road classes [45]. Consequently, strength serves as the primary control parameter in the mix design process. The standard flexural strength values established for ordinary concrete, shown in Table 11, are not applicable to PCNRD due to its distinct structural characteristics [48]. Through extensive experimentation, it has been demonstrated that incorporating admixtures such as silica fume can significantly enhance the strength of PCNRD, allowing its flexural strength to meet both functional and structural requirements [49,50]. Based on the previous research conducted by this group, the standard flexural strength values for PCNRD are presented in Table 12.

When PCNRD is employed as a pavement structural layer, flexural strength serves as the primary design index. However, the preparation of flexural strength specimens is relatively complex, whereas compressive strength testing is more convenient and practical. Therefore, establishing a correlation between flexural and compressive strengths is essential. Experimental data indicate a strong correlation between these two parameters. The regression relationship developed by this research group is expressed in Equation (15), from which the compressive strength values corresponding to the flexural strengths in Table 11 were calculated.

(15) $$f_{f}=0.5001{f_{c}}^{0.6299}(R^{2}=0.9030)$$

where *f_f_* is the 28 d flexural strength (MPa); *f_c_* is the 28 d compressive strength (MPa).

In numerous studies of concrete-/cement-based materials, the *R*^2^ values for compressive and flexural strengths typically range between 0.85 and 0.98 [51,52,53]. For pavement materials, an *R*^2^ ≥ 0.85 is sufficient to support the estimation of flexural strength from compressive strength in design calculations [54]. An R^2^ value of 0.9030 falls toward the upper end of this range, indicating that the model explains approximately 90% of the data variation and possesses strong predictive capability.

(2) Porosity

A high porosity not only facilitates rapid drainage of water from within the pavement structure but also contributes to superior noise reduction performance. However, excessive porosity diminishes the material’s strength, stiffness, and durability, resulting in an inadequate bearing capacity of the surface layer [55]. At present, specifications do not clearly define the required surface porosity. Considering potential uncertainties during construction—such as partial blockage of voids by slurry—and to satisfy performance requirements, the target range of effective porosity is set between 17% and 23%.

#### 3.4.2. Formula Regression Results

The uniform experimental results are shown in Table 13. The collected data were analyzed, and regression analyses were conducted to determine the relationships between four influencing factors and both the mechanical strength and effective porosity of the PCNRD material. As shown in Table 14, the regression equations exhibit acceptable correlation, with R^2^ values ranging from 0.79 to 0.85. As discussed in Section 3.2.2 and Section 3.4.1, these R^2^ values reflect the inherent variability of the experimental data while still indicating reliable predictive capability. During the mix proportion design of PCNRD, based on the predetermined design strength and target porosity, the regression equations provided in Table 15 can be utilized to establish the initial mix proportions.

#### 3.4.3. Procedure of Mix Design

The mix design of PCNRD must ensure that both its mechanical strength and drainage performance meet the required design criteria while maintaining appropriate workability. In this study, a mix design methodology is proposed based on extensive experimental investigations.

(1) Calculation of the initial mix proportion

① Determination of the objective void ratio

As discussed previously, the optimal effective void ratio for the surface layer is determined to range between 17% and 23%. The objective void ratio was determined by meteorological and hydrological data, including local rainfall and temperature. According to the effective void ratio, the full void ratio (*n*_0_) is calculated using regression Equation (12).

② Determination of coarse aggregate content

According to the established relationship shown in Equation (13) between the void ratio and unit volume ratio of coarse aggregate α, α can be back-calculated when the void is known.

Take α into Equation (26) and obtain the coarse aggregate content ($m_{g}$) in unit volume of PCNRD.

(26) $$m_{g}=\rho_{0g}\alpha$$

③ Determination of trial mix strength

To satisfy pavement load-bearing requirements and mitigate variability during production, a safety coefficient must be introduced, as defined by Equation (27).

(27) $${f_{f}}^{\prime }=1.15f_{f}$$

where ${f_{f}}^{\prime }$ is the trial mix flexural tensile strength of PCNRD (MPa).

④ Determination of water–cement ratio

The computational formula for the water–cement ratio, expressed in Equation (28), is derived from the regression Equation (22) based on the uniform experimental analysis.

(28) $$w/c=\frac{f_{f}^{\prime }-0.0415d+1.102}{9.433}$$

⑤ Determination of cement content

The calculation of the void ratio ($P_{c}$), coarse aggregate content ($m_{g}$), and water–cement ratio (w/c) are obtained by the above calculation. The rest of the material consumption can be calculated by the absolute volume method with Equation (29).

(29) $$\frac{m_{c}}{\rho_{c}}+\frac{m_{g}}{\rho_{g}}+\frac{m_{w}}{\rho_{w}}+P_{c}=1$$

The cement content can subsequently be calculated using Equation (30).

(30) $$m_{c}=\frac{\rho_{w}\rho_{c}}{\rho_{w}+w/c\rho_{c}}\left(1-P_{c}-\frac{m_{g}}{\rho_{g}}\right)$$

where $m_{w}$ is the water content in unit volume in concrete, respectively; $\rho_{c}$, $\rho_{g},$ and $\rho_{w}$ are the density of cement, coarse aggregate, and water, respectively (the density of coarse aggregate is the apparent density).

(2) Establishment of the Basic Mix Proportion

According to the material content acquired by the above mix proportion, PCNRD can be trial-mixed. The trial-mixed raw material should be in accordance with material used in the practical engineering. Then, the working performance of PCNRD is evaluated by the workability evaluation method described above. If the working state fails to meet the requirements, the mix proportion should be adjusted using the methods provided in Table 15. When the fresh mixture workability is Grade C, it is recorded as qualified. If qualified, the calculated void ratio of fresh concrete should be measured. If unqualified, the mix proportion should be adjusted again to meet the requirements. By changing the coarse aggregate volume ratio and water–cement ratio, the workability performance and void ratio of PCNRD were adjusted. As a result, the basic mix proportion can be determined.

(3) Determination of lab mix proportion

According to the basic mix proportion, several groups of porous concretes with drainage and noise reduction are prepared, and the specimens of compressive and flexural strength were made. After standard curing, the 7 d compressive strength and 28 d flexural strength are tested. If the strength meets the requirement, this test mix proportion becomes the lab mix proportion; if it does not meet requirement, the admixture content must be adjusted, and the test mix proportion is recounted until the strength meets the requirement.

(4) Conversion working mix proportion

The lab mix proportion is obtained using an aggregate in a natural withering state. In a construction site, the water content of aggregates always varies, so the lab mix proportion needs to be adjusted according to the practical water content of the aggregate. The water content of an aggregate should be deduced from the practical water content of concrete. From these results, the conversion mix proportion is prepared.

## 4. Conclusions

In this study, the sound absorption, drainage capacity, and anti-wear performance of the newly developed PCNRD were comprehensively investigated, and a systematic and innovative mix design method was established. Based on test results of this study, the main conclusions are summarized as follows:

(1) The cement content and silica fume dosage exert significant influence on the internal pore structure and acoustic behavior of PCNRD. The average sound absorption coefficient exceeds 0.2, demonstrating that PCNRD provides excellent noise reduction performance suitable for pavement surface applications.
(2) The permeability coefficient increases with the effective porosity, indicating that PCNRD exhibits superior drainage performance compared with asphalt concrete. The optimal effective porosity range is 17–23%, and the recommended upper pavement thickness is 8–10 cm.
(3) The effective porosity shows a strong linear correlation with the total porosity and coarse aggregate content, offering a reliable quantitative basis for optimizing the mix design parameters for PCNRD.
(4) Increasing the cement content effectively reduces the abrasion loss, which remains below 20%, indicating that PCNRD demonstrates good wear resistance suitable for heavy-traffic pavement applications.
(5) Regression relationships between the strength and porosity were established, and a practical mix design procedure specifically for PCNRD was proposed using the strength and effective porosity as dual control indices, providing technical guidance for its broader engineering implementation.

Future research will focus on analyzing the microstructure of porous cementitious concrete to explore the relationship between the pore size distribution and functional properties of porous concrete. Systematic investigations into the effects of varying pore sizes on noise reduction, abrasion resistance, drainage capacity, and strength will be conducted. This will involve refining the characterization of pore structures to reveal how connectivity and distribution influence material durability and functional properties. Concurrently, optimizing mix designs to regulate pore structures while ensuring adequate mechanical performance will emerge as a key research focus for enhancing the overall performance of porous concrete.

## Figures and Tables

**Table 1:** Materials used in the experiment.

Materials	Properties	Dosage
Cement	Ordinary Portland cement (P.O.42.5). Density: 3.1 g/cm^3^ 28-day compressive strength: 53.5 MPa 28-day bending flexural strength: 8.7 MPa	334 kg/m^3^
Coarse aggregate	Particle size: 5–10 mm, diorite. Apparent density: 2.927 g/cm^3^ Particle size: 10–16 mm, diorite. Apparent density: 2.924 g/cm^3^ The result of screening is listed in Table 2	1731 kg/m^3^
Silica fume	Average particle size: 0.1~0.15 μm Specific surface: 15~27 m^2^/g The chemical component of silica fume is listed in Table 3	6% (relative to the cement content)
Water-reducing agent	High-efficiency water reduction	1.5% (relative to the cement content)

**Table 2:** Passing rate of coarse aggregate (%).

Grading (mm)	16	13.2	9.5	4.75	2.36	1.18	0.6	0.3	0.15	0.075
10–16	100.00	87.93	0.00	0.00	0.00	0.00	0.00	0.00	0.00	0.00
5–10	100.00	100.00	99.30	7.70	0.00	0.00	0.00	0.00	0.00	0.00

**Table 3:** Chemical composition of silica fume (%).

SiO_2_	Al_2_O_3_	CaO	MgO	SO_3_	Fe_2_O_3_	Na_2_O	K_2_O	LOI (Loss on Ignition)
90	0.4	0.8	0.6	—	0.3	0.3	0.8	3.8

**Table 4:** Chemical composition of cement (%).

Cement Type	CaO	SiO_2_	Al_2_O_3_	Fe_2_O_3_	MgO	SO_3_	Na_2_O	K_2_O	LOI (Loss on Ignition)
P.O.42.5	63.51	20.63	5.09	4.28	1.47	2.26	—	—	1.3

**Table 5:** Specimens and variables for tests.

Kind of Test	Specimen Size (mm)	Specimen Number for Each Testing Level	Variable		Notes
Standard specimen molding test	150 × 150 × 150	-	-		At 28 days
Sound absorption test	Φ 95 × 80	11 × 1	Silica fume content (wt.%)	0	Frequency is also the variable At 28 days
				3	
				6	
				9	
			Cement content (kg/m^3^)	285	
				310	
				334	
				360	
			Thickness (mm)	60	
				80	
				100	
Permeability test	150 × 150 × 150	5 × 1	Target void ratio (%)	12	At 28 days
				15	
				18	
				20	
				25	
Wear resistance test	Φ 101.6 × 63.5	4 × 3	Cement content (kg/m^3^)	280	At 28 days
				310	
				334	
				360	

**Table 6:** Experimental conditions.

Kind of Test	Factors	Conditions
Standard specimen molding test	Water–cement ratio (W/C)	0.3
	Molding method	Upper vibration molding
Permeability test	Test temperature	10 °C

**Table 7:** Classification of fresh porous concrete workability.

W/C	Workability State	Estimation Scale
Small	Complete disintegration; aggregates are loose and surface lacks luster	A
↓	Partial disintegration; aggregates dull, no surface sheen	B
	Specimen retains the container’s shape; aggregates exhibit surface sheen	C
	Specimen collapses slowly; aggregate surfaces fully glossy	D
Large	Specimen collapses immediately; slurry leakage observed	E

Note: The symbol “↓” indicates that the workability grade increases from small to large.

**Table 8:** Uniform design factors level table of PCNRD mix design test.

Level	Factor			
	A Water–Cement Radio	B Cement Content (kg/m^3^)	C Silica Fume Content (%)	D Strength Grade of Cement (MPa)
1	0.28	285	0	32.5
2	0.30	310	3	42.5
3	0.33	334	6	52.5
4	0.36	360	9	—

**Table 9:** Mixture ratio design table of PCNRD.

Serial Number	Test Number	Mixture Ratio			
		A	B (kg/m^3^)	C (%)	D (MPa)
1-1	1	0.28	334	6	52.5
1-2	2	0.28	285	0	42.5
1-3	3	0.28	334	9	32.5
2-1	4	0.30	285	3	32.5
2-2	5	0.30	334	0	52.5
2-3	6	0.30	285	6	42.5
3-1	7	0.33	360	3	42.5
3-2	8	0.33	310	9	32.5
3-3	9	0.33	360	6	52.5
4-1	10	0.36	310	0	52.5
4-2	11	0.36	360	9	42.5
4-3	12	0.36	310	3	32.5

**Table 10:** Average sound absorption coefficient under different mix proportions.

Mix Proportion	Silica Fume Content (%)				Cement Content (kg/m^3^)			
	0	3	6	9	285	310	334	360
Sum average	0.35	0.23	0.36	0.28	0.33	0.28	0.28	0.33
Arithmetical mean	0.34	0.27	0.30	0.22	0.35	0.3	0.28	0.31
Void ratio (%)	23.4	21.3	19.8	17.3	22.1	20.5	18.6	16.3

**Table 11:** Standard values of flexural strength for ordinary concrete.

Traffic Classification	Ponderosity, Extra Heavy	Heavy	Medium	Light
Flexural strength standard values (MPa)	≥5.0	≥5.0	4.5	4.0

**Table 12:** Flexural strength standard values of PCNRD.

Traffic Classification	Ponderosity, Extra Heavy	Heavy	Medium	Light
The flexural strength standard values of PCNRD (MPa)	≥4.5	≥4	3.5	3.5
The compressive strength standard values of PCNRD (MPa)	≥32	≥27	21	21

**Table 13:** Uniform experimental results.

Serial Number	Test Number	7 d Compressive Strength (MPa)	28 d Flexural Strength (MPa)	Effective Porosity (MPa)
1-1	1	17.5	3.23	19.9
1-2	2	12.8	2.65	22.4
1-3	3	18.3	3.46	18.3
2-1	4	13.1	2.80	22.2
2-2	5	19.1	3.95	18.9
2-3	6	16.7	3.28	20.4
3-1	7	21.5	4.30	17.1
3-2	8	19.6	4.16	17.9
3-3	9	22.0	5.27	18.9
4-1	10	18.4	4.24	18.9
4-2	11	17.5	3.59	19.0
4-3	12	14.4	2.95	21.1

**Table 14:** Regression equation of PCNRD.

Dependent Variable	Independent Variable	Regression Equation	Serial Number
$f_{c,7}$	w/c, c, s, d	$f_{c,7}=6.950w/c+0.057c+0.182s+0.102d-8.125$ $\left(R^{2}=0.801\right)$	(16)
	w/c, s, d	$f_{c,7}=21.926w/c+0.434s+0.210d-0.273$ $\left(R^{2}=0.841\right)$	(17)
	c, s, d	$f_{c,7}=0.061c+0.168s+0.095d-6.703$ $\left(R^{2}=0.790\right)$	(18)
	c, s	$f_{c,7}=-6.992+0.0757c+0.041s$ $\left(R^{2}=0.861\right)$	(19)
$f_{f}$	w/c, c, s, d	$f_{f}=6.782w/c+0.010c+0.035s+0.035d-3.249$ $\left(R^{2}=0.828\right)$	(20)
	w/c, s, d	$f_{f}=9.327w/c+0.078s+0.053d-1.914$ $\left(R^{2}=0.817\right)$	(21)
	w/c, d	$f_{f}=-1.102+9.433w/c+0.0415d$ $\left(R^{2}=0.811\right)$	(22)
$n_{e}$	w/c, c, s, d	$n_{e}=-5.544w/c-0.035c-0.086s-0.005d+33.370$ $\left(R^{2}=0.744\right)$	(23)
	w/c, s, d	$n_{e}=-14.862w/c-0.243s-0.073d+28.485$ $\left(R^{2}=0.852\right)$	(24)
	c, s	$n_{e}=-0.038c-0.074s+32.237$ $\left(R^{2}=0.837\right)$	(25)

where $f_{c,7}$ is the 7 d compressive strength of PCNRD (MPa); *c* is the cement content (kg/m^3^); *s* is the silica fume content (%); *d* is the strength grade of cement (MPa).

**Table 15:** Adjustment method of workability and void ratio of PCNRD.

Items	Objective Value	Adjustment Method
Void ratio	*P_c_*	Exceed objective value and reduce unit volume ratio of coarse aggregate. Be inferior to objective value and increase unit volume ratio of coarse aggregate.
State grade of workability	C	Grade A and B is to increase water–cement ratio; Grade C and D is to reduce the water–cement ratio.

## Data Availability

The original contributions presented in this study are included in the article. Further inquiries can be directed to the corresponding author.

## Abbreviations

The following abbreviations are used in this manuscript:

PCNRD	porous concrete with noise reduction and drainage performance
W/C	water–cement ratio
LOI	loss on ignition
